# Hæmoptysis and Tubercle

**Published:** 1852-02

**Authors:** 


### SELECTIONS

FROM

### FOREIGN AND HOME MEDICAL JOURNALS.

From Dr. Walshe's admirable work on *Diseases of the Lungs and Heart*, we copy the following:

Hæmoptysis may occur, *mechanically*, from diminished pressure of the atmosphere, as in the ascent of lofty mountains, and from injuries of various kinds to the chest; and it may take place *vicariously*, as a periodical discharge in females, instead of the catamenia; unless, under these circumstances, it is a sign of *disease of the lungs, heart, or great vessels.* The diseases which act as more or less frequent causes of hæmoptysis, are tubercle, cancer, aneurism, cardiac diseases, and certain affections of the larynx, trachea, and bronchial tubes. In actual clinical practice, hæmoptysis is so frequently connected with tuberculous disease, that it comes to be one of its most significant symptoms. The laws of tuberculous hæmoptysis will hereafter be considered. In the present place, I shall simply place before the reader an analysis of the clinical evidence I have been able to collect on the question of the connexion of hæmoptysis with tubercle *in adults.*

The *quantity* of blood voided, is the first point of consideration. It is commonly said that the expectoration of streaked or tinged sputa is utterly insignificant, because such are seen in *bronchitis*, &c., &c.; but no attempt has ever been made to decide numerically to what extent this is true. I find that in twenty-five cases (observed at Brompton, and at University College Hospital) of chronic bronchitis with or without marked emphysema, (*but always without serious disease of the heart,*) the absence of such expectoration was noted in nineteen cases, its presence in six. Now in *all* these six cases of streaked expectoration, there was more or less ground for suspicion that tubercles were to a slight amount present,—in two of them this was proved to be the fact by *post-mortem* examination. While, then, as I have found, bloody expectoration occurs in 71.79 per cent. of tuberculized persons in the first stage, it occurred in 24 per cent. of bronchitic people (free from serious cardiac disease;) but in all of the latter there was either suspicion or certainty of the existence of tubercle to a *slight* amount.* The mean duration of the disease in the phthisical cases 26.55 months, in the bronchitic, 49.50,—hence the significance (*quoad* tubercle) of hæmoptysis is greater even than the relative per-centages above given would signify. Streaked or tinged sputa are rarely or never the 'first symptom' of phthisis; should they appear in this guise, then, they would probably be dependent on some other cause. The question of hæmoptysis in plastic bronchitis has already been referred to.

*Primary cancer of the lung and medianastina*, as I have elsewhere shown, from the analysis of a small number of recorded cases, is very frequently attended with sanguineous expectoration of pure hæmoptysis. In regard of this symptom, the two diseases may be thus compared: the per-centage of hæmoptysis *of all amounts* in cases of cancer is 72, in phthisis 80 92; while hæmoptysis *above one ounce* occurs in cancer and phthisis in the ratio of about 70 to 40. Hence 100 cases of cancer of the lung will be attended nearly as often with hæmoptysis of all amounts, and greatly more often with hæmoptysis above an ounce in amount at a time, than 100 eases of phthisis. But, on the other hand, tuberculous is so vastly more frequent than cancerous disease of the lung, that the share of the

* "Pathologically, these people were *latently* tuberculous persons, with superadded bronchitis; but practically, they could only be regarded as bronchitic."

population suffering at any time from cancerous hæmoptysis will form but an insignificant fraction of that suffering from hæmoptysis of tuberculous origin.

It is not sufficiently insisted upon by writers, that *empyema* does not give rise to hæmoptysis. In sixteen well marked cases which I have had under my care within the last two years, for periods of three months and upwards, no single example of hæmoptysis occurred.—But more than this, empyema, established in a phthisical person, seems to be *a preventive of hæmoptysis.* In seven cases of combined tuberculous excavation and empyema, carefully watched, and proved as to the diagnosis, either by dissection or by indubitable signs, no spitting of blood had ever occurred. All these seven persons were males. The pressure exercised on the lung by the contents of the pleura might appear to explain the fact plausibly enough, especially as the excavations were on the same side as the the empyema in six of the seven cases; and I have recently seen a curious exemplification of the apparent influence of excessively acute pleuritic effusion in arresting obstinate hæmoptysis. But, on the other hand, in one case, excavations existed in both lungs.

*Simple chronic consolidation of the lung* has not, in my experience, been attended with hæmoptysis to any amount.

*Acute pneumonia,* accompanied with a discharge of pure blood, is almost positively connected with tuberculous disease.

*Gangrene* of the lung is rarely attended with hæmoptysis in the adult; in infancy (when tuberculous hæmoptysis is very rare) it is rather common.

I have never known *ulceration of the larynx* productive of discharge of blood to any extent; streaks are not uncommon. But ulceration of the larynx (proceeding from within outwards) seems not to occur as a primary affection; at least I have never seen it except in phthisis (which may be completely latent in regard of the lung,) cancer, and syphilis.

Hæmoptysis arising from *disease of the heart* can with difficulty be confounded, even in itself, with the severer forms of phthisical hemorrhage; while physical signs of the cardiac disease will point to its true source in such cases of the slighter form. I have never once seen cardiac disease, of such kind as to cause hæmoptysis, co-existent with *phthisis*, using the term in its practical sense; but in a fair number of instances I have seen advanced cardiac disease in persons whose lungs contained *crude tubercles and gray granulations.* It may be, therefore, that the conditions of the system existing in heart-disease are unfavorable to the development of tubercle; but the infrequency with which the two kinds of disease are found together doubtless depends, in the main, on the difference in the periods of life at which each is especially prone to occur.

*Aortic aneurism*, opening into the trachea, may (without proving immediately fatal) give rise to hemorrhage, indistinguishable by its own characters from profuse pulmonary hemorrhage. The history of the case, the physical signs, the age of the individual, &c., commonly establish the diagnosis; but when the aneurism is small, and so placed as to elude percussion, and pressure-signs, both concentric and eccentric, are absent, the difficulty of *proving* the existence of aneurism may be insurmountable; the existence of the disease may be divined, but not demonstrated. It is to be remembered that the absence of notable signs of tuberculization does not justify the inference that the hæmoptysis is not phthisical, seeing that a tremendous pulmonary hemorrhage may occur when slight consolidation exists at one apex only, and that such consolidation might be supposed to depend on the pressure of an aneurism.

It is matter of common belief that in women who menstruate imperfectly and irregularly, the expectoration of a small quantity of blood is insignificant. I think this *perhaps* true where streaks only are concerned; but in every instance I have observed (*except one*,) where such succedaneous hæmoptysis reached an ounce or upwards, there has been either evidence, or ground for suspicion, of tuberculization. Similarly I have seen two cases of individuals presumedly in a state of perfect health, who in a violent fit of passion brought up a certain quantity of blood from the lungs: *both had latent tubercles.*

The tendency of my experience, then, is clearly to show the vast frequency with which hæmoptysis is in some manner or other an attendant on tuberculous disease.—The fact, that individuals are occasionally met with who, after having had more or less profuse hæmoptysis, live on to a good age without exhibiting phthisical symptoms, does not invalidate this result; it simply shows that tuberculization tending to hæmoptysis may, as well as that not so tending, undergo spontaneous suspension.

The *diagnosis* of hæmoptysis in general may be considered in the present place; while we reserve for future consideration any special points connected with its distinction, in particular diseases of the lungs and heart.

Hemorrhage from the mouth and fauces can be distinguished by careful inspection of these parts from hæmoptysis. Epistaxis, under ordinary circumstances, cannot be confounded with hæmoptysis; but sometimes blood, instead of coming forwards from the nares, trickles backwards, and may be from time to time coughed up. But here, again, close examination of the nares anteriorly, and of the pharynx, will disclose the source of the hemorrhage.

Hæmoptysis is distinguished from hæmatemesis by the following characters. Hæmoptysis is preceded by slight dyspnœa, anomalous sensations about the chest, tightness, weight behind the sternum, or at some other spot, to which the patient will sometimes confidently point as the seat of mischief; hæmatemesis by weight and uneasiness as the epigastrium, sometimes by nausea. When the lung supplies the blood, the pulse is oftener excited, full, bounding, sometimes *bis feriens,* than when the stomach is its source. A salt taste in the mouth, with tickling and gurgling sensations in the throat, often precedes actual hæmoptysis; whereas I certainly have never known this complained of in hæmatemesis. Blood is ejected in severe hæmoptysis with efforts indistinguishable by patients from those of true vomiting; but previously to such "vomiting of blood," mouthfuls have generally been coughed up. Hæmatemesis is attended and followed by tenderness at the epigastrium; hæmoptysis by none of this. No matter what quantity of blood be brought up from the lungs at once, small quantities continue to be expectorated foı a time; when the stomach is at fault, on the contrary, full discharge occurs suddenly, and there is, generally speaking, an end of the matter,—certainly no bloody or stained sputa follow. In hæmoptysis the blood is florid and frothy; in hæmatemesis dark and non-aerated: at least this is the common case. But when large masses of blood are discharged from the lungs, they may be toally frothless; and where hemorrhage occurs rapidly from an artery in the stomach, the blood is vomited at once, and of perfectly arterial hue: no time is allowed for discoloration by the gastric fluids. Discharge of blood by stool is the rule in hæmatemesis; the exception in hæmoptysis: in the latter case, it comes of blood accidentally swallowed, and is never, so far as I have known, abundant. In hæmoptysis liquid rhonchus may almost invariably be found in some part of one or both lungs; nothing of the sort exists in hæmatemesis; in the former, the pulse is proportionably less quickened than the respiration; still this perversion may occur in hæmatemesis also. Lastly, the diagnosis should never be fixed on without inquiring into the history of the patient, and making a cautious examination of the chest. Should the evidence of chronic changes at the apices exist, a doubtful opinion in favor of pulmonary origin would at once be strengthened; but the absence of such changes would not exclnde the possibility of hæmoptysis; for, as will hereafter fully appear, such discharge of blood may occur before any notable physical changes have occurred in the lungs. The state of the chylopoietic viscera should be examined physically in aid of the diagnosis of hæmatemesis.

*Diagnosis of Phthisis.*—I propose, instead of systematically considering the *diagnosis* of chronic phthisis, to embody in a series of propositions, some of the most interesting facts, of which I have actually seen illustrations.

A young adult, who has had an obstinate cough, which commenced without coryza and without any very obvious cause, a cough at first dry and subsequently attended for a time with watery or mucilaginous-looking expectoration, and who has wandering pains about the chest, and loses flesh even slightly, is in all probability phthisical. If there be hæmoptysis to the amount of a drachm even, the diagnosis becomes, if the patient be a male and positively free from aneurism and mitral disease, almost positive. If, in addtion, there be slight dulness under percussion at one apex, with jerking or divided and harsh respiration, while the resonance at the sternal notch is natural, the diagnosis of the first stage of phthisis becomes next to absolutely certain. But not absolutely certain: for I have known every one of the conditions in *a*, *b*, and *c* exist (except hæmoptysis, the deficiency of which was purely accidental,) when one apex was infiltrated with encephaloid cancer, and no cancer had been discovered elsewhere to suggest to the physician its presence in the lung. If there be cough, such as described, and permanent weakness and hoarseness of the voice, the chances are very strong (provided he be non-syphilitic) that the patient is phthisical. If decidedly harsh respiration exist at the left apex or at the right apex behind, if the rhythm of the act be such as I have called *cogged-wheel*, and there be dulness, so slight even as to require the dynamic test for its discovery, there can be little doubt of the existence of phthisis. If with the same combination of circumstances deep inspiration evokes a few clicks of dry crackling rhonchus, the diagnosis of phthisis, so far as I have observed, is absolutely certain. If these clicks on subsequent examination grow more liquid, the transition from the first to the second stage may be positively announced. If there be slight flattening under one clavicle, with deficiency of expansion-movement, harsh respiration and slight dulness under percussion, without the local or general symptoms of phthisis, the first stage of tuberculization cannot be diagnosticated with any surety, unless there be incipient signs at the other apex also: the conditions in question limited to one side might depend on chronic pneumonia or on thick induration-matter in the pleura.—The existence of limited, though marked, dulness under one clavicle, with bronchial respiration and pectoriloquy, so powerful as to be painful to the ear, the other apex giving natural results, will not justify the diagnosis of phthisis. I have known this combination when the apex of the lung was of model health, and a fibrous mass, the size of a walnut, lay between the two laminæ of the pleura. I would even go further, and say that the combination in question is rather hostile than otherwise to the admission of phthisis; as, had tuberculous excavation formed at one side, the other lung would, in infinite probability, have been affected in an earlier stage. Pneumonia, limited to the supra- and infra-clavicular region on one side, and not extending backwards, is commonly, but not always, tuberculous. Subscrepitant rhonchus, limited to one base posteriorly, is not, as has been said, peculiar to tubercle; it may exist in emphysema and in mitral disease. Chronic peritonitis, in a person aged more than fifteen years, provided cancer can be excluded, involves as a necessity the existence of tubercles in the lungs. To this law of Louis's, it is necessary to add the qualification, provided Bright's disease be also absent. Pleurisy with effusion, which runs a chronic course in spite of ordinary treatment, is, in the majority of cases, tuberculous or cancerous: the character of the symptoms, previously to the pleurisy, will generally decide between the two.—Double pleurisy, with effusion, is not, as has been said, significant of tubercle; for it may depend on Bright's disease. If the latter disease can be excluded, carcinoma and pyohæmia remain as other possible causes. If a young adult, free from dysentery, and who has not resided in tropical climates, suffers from obstinate diarrhea, which goes on month after month, with slight remissions or intermissions, even though there be no cough, he is in most strong probability phthisical. If physical signs, to the slightest amount, exist at either apex, he is, almost to absolute certainty, phthisical. If a young adult, free from secondary syphilis and spermatorrhœa, and not dissolute in his habits, steadily lose weight, without clear cause, he is in all probability phthisical, even though no subjective chest-symptoms exist. But he is not by any means certainly so; for he may have latent cancer in some unimportant organ, or he may have chronic pneumonia.—Nay, more, he may steadily lose weight, have dry cough, occasional diarrhea, and night-sweats, and present dulness under percussion, and bronchial respiration under both clavicles, and yet be non-phthisical. I have known all this occur in cases, both when the lungs were infiltrated superiorly with primary encephaloid cancer, and when they contained secondary nodules of the same kind.—Failure of weight becomes less valuable as a sign of phthisis, the longer the thirtieth year has been passed. The discovery of cardiac disease with marked symptoms deposes against, but does not exclude, the existence of active tuberculization. The existence of cancer in any organ is unfavorable to the presence of tuberculous disease; but tubercle and cancer *may* co-exist, even in the same lung.

I would here add, for the sake of beginners, a few cautions in the applications of physical diagnosis in phthisis. Never attempt to give a positive opinion as to the actual state of the lungs, where there has been recent hæmoptysis, or when pleuritic effusion, bronchitis, or pneumonia is present: I of course refer to cases where there may or may not be signs of the first stage; if excavation exist, its signs may be unravelled positively in spite of these complications. Trust very little, if at all, to the conditions of vocal resonance; accept with great caution the evidence of slight changes in respiration, unless they be corroborated by percussion-changes; place no confidence in jerking respiration (even though local) in a hysterical woman,—nor in harsh respiration limited to the right apex in any woman, nor in very slight dulness at the right front apex in man or woman. Lastly, never give a confident opinion, in a nicely-balanced case, from a single examination; and make examinations in various postures.

